# Increase in testosterone and cortisol one week after repeated exercise in a cold environment

**DOI:** 10.3389/fphys.2025.1731242

**Published:** 2026-01-13

**Authors:** Jana Jaklová Dytrtová, Michal Jakl, Radim Jebavý, Ludmila Máčová, Daniela Horníková, František Novák, Petr Vodička, Tomáš Navrátil, Marie Bičíková, Barbara Elsnicová, Jitka Žurmanová, František Galatík

**Affiliations:** 1 Faculty of Physical Education and Sport, Sport Sciences–Biomedical Department, Charles University, Prague, Czechia; 2 Faculty of Physical Education and Sport, Department of Athletics, Charles University, Prague, Czechia; 3 Institute of Endocrinology, Prague, Czechia; 4 Faculty of Science, Department of Physiology, Charles University, Prague, Czechia; 5 4th Department of Internal Medicine, First Faculty of Medicine, General University Hospital in Prague, Charles University, Prague, Czechia; 6 Institute of Animal Physiology and Genetics, Czech Academy of Sciences, Libechov, Czechia; 7 J. Heyrovsky Institute of Physical Chemistry, Czech Academy of Sciences, Prague, Czechia

**Keywords:** androgens, cold acclimation, cold exposure, cortisol, exercise, testosterone

## Abstract

**Introduction:**

Effects of cold exposure on human physiology are mainly studied after exercise. Therefore, this study aims to investigate the effects of gradually increasing cold exposure and physical exercise on steroid levels, body composition, and other biochemical markers in healthy male athletes immediately after 5-day exercise in cold and after 7 days of recovery.

**Methods:**

Healthy male athletes (n = 12, aged 20.5 ± 1 year, height 181 ± 7.7 cm) were exposed to 5 days of outdoor physical training (2 °C–3 °C) with increasing intensity of exercise and cold exposure. Venous blood was collected, and body bioelectrical impedance measured before and after the 5-day experiment, and after 7-day recovery. Circulating levels of testosterone, cortisol, androstenedione, dehydroepiandrosterone sulphate, 17-hydroxyprogesterone, calcifediol, interleukin-6, C-reactive protein, and erythrocyte superoxide dismutase activity were analysed.

**Results:**

Our data show a delayed effect of exercise in cold after 7 days of recovery in the total plasma levels of testosterone (56% increase vs. baseline) and cortisol (54% increase vs. baseline), with no difference immediately after physical training in cold. Bioelectrical impedance analysis showed a decrease in waist-to-hip ratio after the experiment, which normalised after 7 days. No significant changes were observed in Interleukin-6, C-reactive protein, or superoxide dismutase levels.

**Conclusion:**

A 5-day period of daily exercise in a cold environment showed no immediate effects, but a potential to elicit adaptive changes delayed for up to 7 days, leading to a significant increase in steroid hormones, without changing the testosterone/cortisol ratio.

## 1 Introduction

In recent years, deliberate cold exposure, e.g., cold plunge or cold-water immersion (CWI), has risen in popularity among both professional and recreational athletes (Ihsan et al., 2021). Research on CWI shows mixed outcomes: it may improve short-term recovery of power output and subjective fatigue after high-intensity exercise, but can also blunt anabolic signalling and adaptations following resistance training (Roberts et al., 2015; Petersen and Fyfe, 2021; Moore et al., 2022). Overall, post-exercise cold exposure appears to influence recovery and adaptation in a manner highly dependent on training type, duration, temperature, and timing. Much less is known about the physiological effects of cold exposure during exercise rather than after it. Changes in steroid hormones are of particular interest due to their impact on recovery, strength, and muscle development.

This study explored the effects of progressively increased physical strain and cold exposure during training on body parameters, focusing on changes in steroid hormones. Testosterone is the most important natural anabolic steroid. In skeletal muscle it promotes proteosynthesis by both genomic and non-genomic action (Mauras et al., 1998; Estrada et al., 2003; Norman et al., 2004). Endogenous testosterone can be synthesized through different pathways. The human body prefers the conversion of prognenolone into dehydroepiandrosterone (DHEA) and further into androstenedione which is finally metabolised into testosterone. Both DHEA and androstenedione have weaker anabolic effects (Dehennin et al., 2001). DHEA is stored in its inactive sulphated form DHEA-S. Due to its much longer half-life and lower clearance, circulating levels of DHEA-S are much higher and show very limited diurnal changes (Kroboth et al., 1999). Alternative synthesis of androstenedione and therefore testosterone involves 17-hydroxyprogesterone (17(OH)Progesterone) which is also a precursor of cortisol. Cortisol is a stress-related steroid hormone, it induces catabolism which is intended to free up resources for proteinogenesis and recovery of homeostasis after stressful events such as exercise or cold exposure (Hackney and Walz, 2013).

Since both exercise and cold exposure can be considered a type of stress, they can also influence the immune and antioxidant systems. Bridging the gap between steroids and the immune system is vitamin D. Vitamin D is essential in bone growth an health, but also has a wide variety of immune effects including improvement of immunity against patogens and reduction of autoimmune responses (Daryabor et al., 2023). Calcifediol is a less active form of vitamin D and a precursor of the most active form calcitriol. Due to calcifediol’s long half-life (20–24 days) it is an excellent marker of overall vitamin D status and storage (Gil et al., 2018). Acute endurance exercise has been shown to increase calcifediol levels in active males (Mieszkowski et al., 2020; Dzik et al., 2022). Additionally supplementation of calcifediol has a potential to improve strength in individuals with muscle weakness but less so in healthy people (Barbagallo et al., 2022). The cytokine Interleukin-6 (IL-6) is another important molecule involved in both acute and chronic phases of inflammation. During exercise, IL-6 can be acutely produced by skeletal muscle to elevate its plasma concentration more than 100-fold. During exercise IL-6 increases the translocation of glucose transporter GLUT4 to sarcolemma and facilitates a crosstalk between skeletal muscle and other organs, such as liver where it was shown to increase glucose and triglyceride production. Conversely, resting levels of IL-6 decrease with long-term regular exercise. The resting IL-6 is mainly produced by immune cells and adipose tissue rather than muscle and is therefore associated with overall inflammatory status. People practicing long-term exercise programs have been shown to have chronically lower levels of resting IL-6 as well as C-reactive protein (CRP) and other markers of inflammation (Nash et al., 2023). Additionally, a single bout of exercise was shown to increase the production of reactive oxygen species. This type of stress is combated by innate antioxidants (Muthusamy et al., 2012). Due to erythrocytes being the most abundant cells in blood and the close contact with oxygen, their antioxidant defence is very important. The main erythrocyte antioxidant is the copper-zinc superoxide dismutase (Cu-Zn SOD) which is a cytosolic enzyme that catalyses the dismutation of O_2_^•–^ into O_2_ and H_2_O_2_ (McCord and Fridovich, 1969; Noor et al., 2002). While studying professional cyclists it has been shown that Cu-Zn SOD activity in erythrocytes increases in chronic aerobic training compared to sedentary individuals, but brief exercise produced no change in sedentary individuals (Mena et al., 1991). Additionaly, in mice SOD1 was shown to be essential for erythrocyte longevity and suppression of autoimmune inflammation (Iuchi et al., 2007).

We were interested in whether the addition of cold temperature could interfere with these previously observed reactions to physical exercise in healthy male athletes. Therefore, we investigated the effect of repeated exercise in a cold environment on circulating levels of total testosterone, cortisol, and their precursors as well as body composition and inflammation/oxidative stress markers. Changes in these metabolites are typically measured shortly after the experimental event, with the understanding that under normal conditions, these changes are transient and short-lived. We aimed to observe how the effects will evolve after a 7-day post-experiment recovery period, which, to our knowledge, was not previously investigated and could bring novel information about the adaptation to the stress of exercise in cold.

## 2 Materials and methods

### 2.1 Participants

The research group consisted of 12 healthy men, aged 20.5 ± 1 year, height 181 ± 7.7 cm. Before the experiment, medical history was taken and an initial medical examination was performed. The exclusion criteria were chronic illness or signs of acute illness. There was no dedicated control group; the whole group was measured at baseline to gather reference data, and repeated measurements were performed on the same individuals throughout the experiment. All participants were of above-average fitness for this age group, with total body fat around 10% of body weight and total muscle mass ≥50% of body weight. Average body composition parameters (InBody) of all participants before the experiment are shown in Table 1. All experiments were approved by the institutional ethical board of the Faculty of Physical Education and Sport, Charles University (no. 129/2023), and all participants provided written informed consent.

**TABLE 1:** Body parameters before and throughout the experiment. Exercise + cold (EC), exercise + cold recovery (ECR), body mass index (BMI), visceral fat area (VFA), and extracellular water to total body water (ECW/TBW). Values are mean. *p < 0.05 vs. baseline. 95% confidence interval of the mean (CI). Repeated measures one-way ANOVA with Tukey’s multiple comparison test.

Parameter	Baseline	95% CI	EC	95% CI	ECR	95% CI
Body weight (kg)	77.7	72.6–82.9	77.5	72.2–82.8	77.6	72.2–83.0
BMI (kg/m2)	23.4	22.4–24.4	23.3	22.2–24.4	23.3	22.2–24.4
Total fat content (kg)	8.6	6.4–10.8	8.2	6.1–10.4	8.5	6.4–10.7
Relative fat content %	11.0	8.3–13.7	10.6	7.9–13.2	10.7	8.0–13.4
VFA (cm^2^)	31	21–42	29	20–40	30	20–40
Waist to hip ratio	0.800	0.780–0.820	0.790*	0.771–0.810	0.790	0.772–0.810
Skeletal muscles (kg)	39.7	36.9–42.4	39.7	36.8–42.7	39.7	36.8–42.6
Total protein (kg)	13.8	12.9–14.7	13.8	12.8–14.8	13.8	12.8–14.8
Intracellular water (L)	32.0	30.0–34.0	32.0	30–34	32.0	30.0–34.0
Extracellular water (L)	18	29.8–34.1	18.7	29.7–34.2	18.5	29.8–34.2
Total minerals (kg)	4.76	4.43–5.08	4.78	4.43–5.12	4.78	4.42–5.14
Skeleton (kg)	3.92	3.64–4.19	4.17	3.64–4.69	3.95	3.65–4.25
Physical fitness score	86.0	82.1–89.9	85.9	81.6–90.2	86.0	81.9–90.1
ECW/TBW	0.37	0.36–0.37	0.37	0.37–0.37	0.37	0.36–0.37

### 2.2 Physical exercise in a cold environment

The experimental protocol was conducted in winter in a mountain environment at 1,000 m a.s.l. (Giant Mountain National Park, Czechia). Baseline blood sampling and body-composition measurements were performed on site under identical ambient conditions. All participants completed a 5-day structured program of combined physical exercise and cold exposure (EC) followed by 7 days of recovery under standard environmental conditions (ECR). To improve clarity and reproducibility, the EC protocol is described below (Table 2) in a strict chronological timeline, with each day summarising (i) primary physical-exercise load and (ii) cold-exposure procedures.

**TABLE 2:** Overview of the 5-day exercise and cold-exposure protocol (EC).

Day	Physical activity (type and duration)	Intensity	Cold acclimation (method and duration)	Environmental parameters
1	Running 30 min (aerobic zone)	Aerobic → mixed	–	3 °C, light clothing
	Running 30 min (mixed zone)			
2	Cross-country (XC) skiing 2 h (mixed zone)	Mixed → near anaerobic	Morning fasted run in swimming trunks (5 min)	2 °C, mild breeze
	Running 1 h (mixed → anaerobic threshold)		Breathwork + squats/push-ups (5 min)	
	Strength endurance 45 min			
3	Winter hiking 8 h (incl. 2 h exposed terrain with strong wind ∼100 km/h)	Aerobic prolonged load	Morning fasted run in swimming trunks (7 min) Breathwork + squats/push-ups (4 min) Evening cold-water immersion to waist (2 min at 2 °C)	2 °C, moderate wind; strong wind exposure during hiking
4	XC skiing 3 h (aerobic → mixed)	Aerobic → mixed; high-intensity intervals	Morning fasted run in swimming trunks (7 min)	2 °C, moderate wind
	Speed-endurance running/XC skiing 90 min		Breathwork + squats/push-ups (3 min)	
	Strength endurance 45 min		Barefoot walk in cold stream, knee depth (2 min)	
5	XC skiing 45 min (aerobic + anaerobic)	Mixed → anaerobic	Morning fasted run in swimming trunks (3 min)	2 °C, moderate wind
	Running 45 min (aerobic + anaerobic)		Breathwork barefoot on snow (2 min)	
			Cold-water immersion lying on back/front (1 min at 2 °C)	

### 2.3 In body – body composition assessment

Body composition was assessed using the InBody 770 (InBody Co., Ltd., Seoul, Republic of Korea), a multifrequency bioelectrical impedance analyser. Measurements were conducted on-site at the venue of the intervention, before the first and after the last day of the programme (a 5-day interval). Participants were instructed to undergo the assessment wearing only their underwear to ensure accuracy and consistency of the results.

### 2.4 Blood collection

From the pre-experiment phase, after EC, and after ECR, blood samples from individual probands were taken in the morning, on an empty stomach, in a faculty room equipped for this purpose. Drinking water was provided for hydration. Two samples of whole peripheral venous blood were collected by a trained medical professional into serum (with micronised silicate particles, no anticoagulant) and plasma (treated with heparin) test tubes (VACUETTE®). After 15 min, the whole and clotted blood were centrifuged (2,000 × g for 15 min). Plasma and serum supernatants were then aliquoted and frozen to −80 °C until further analysis.

### 2.5 Analysis of inflammation markers

Plasma interleukin-6 (IL-6) was determined by chemiluminescence immunoassay (CLIA) using an automated analyser DXI Beckman Coulter (IL-6 reference values <6.4 ng/L). Serum C-reactive protein (CRP) was determined by the immunoturbidimetric method using an automated analyser DXI Beckman Coulter (CRP reference values <0.5 mg/L).

### 2.6 Analysis of circulating hormones

Collected serum samples were used for the detection of hormones using liquid chromatography tandem-mass spectrometry (LC-MS/MS) methods. The analysis of testosterone, cortisol, androstenedione, DHEA-S, and 17(OH)Progesterone was performed using IVD-certified ClinMass® LC-MS/MS Complete Kit Steroids in Serum/Plasma from RECIPE (RECIPE Chemicals + Instruments GmbH, München, Germany). Calcifediol was measured according to Bičíková et al. (2022).

### 2.7 ELISA assay of erythrocyte SOD activity

Erythrocytes separated from clotted blood were lysed in four times their volume of ice-cold HPLC-grade water and centrifuged at 10,000 × g for 15 min at 4 °C. The supernatant (erythrocyte lysate) was then analysed using a Superoxide Dismutase ELISA kit (Cayman Chemical, 706002) following the manufacturer’s instructions.

### 2.8 Statistical analysis

Statistical analyses were conducted using the GraphPad Prism 8 software (GraphPad, San Diego, CA). The distribution of data was analysed by the Shapiro–Wilk and Kolmogorov–Smirnov normality tests. The identification of outliers was carried out using the ROUT (Robust regression and Outlier removal) method with a ROUT coefficient set at Q = 1%. For parametric data, repeated measures one-way ANOVA with Tukey’s multiple comparison test was employed to identify significant differences between the means of individual groups. For nonparametric data, the Friedman test with Dunn’s multiple comparisons test was used. The significance level of p ≤ 0.05 was considered statistically significant. Data are presented as means with 95% confidence interval (CI). To visualize the overall levels of analysed hormones between baseline and ECR, the values were scaled and centred, and the heatmap was constructed using the R package ComplexHeatmap (Marvanova et al., 2023).

## 3 Results

### 3.1 Characterization of the experimental group and analysis of body composition after exercise in a cold environment

InBody measurements of body composition offered characterization of the experimental group before the experiment. We did not observe any noteworthy changes during the course of the experiment. The only significant change was in waist to hip ratio (p = 0.049), which decreased slightly (−1.25%) post EC compared to baseline, this lower mean persisted into ECR but became statistically insignificant (p = 0.15) compared to baseline (Table 1).

### 3.2 Analysis of hormonal changes after exercise in a cold environment

**FIGURE 1:**
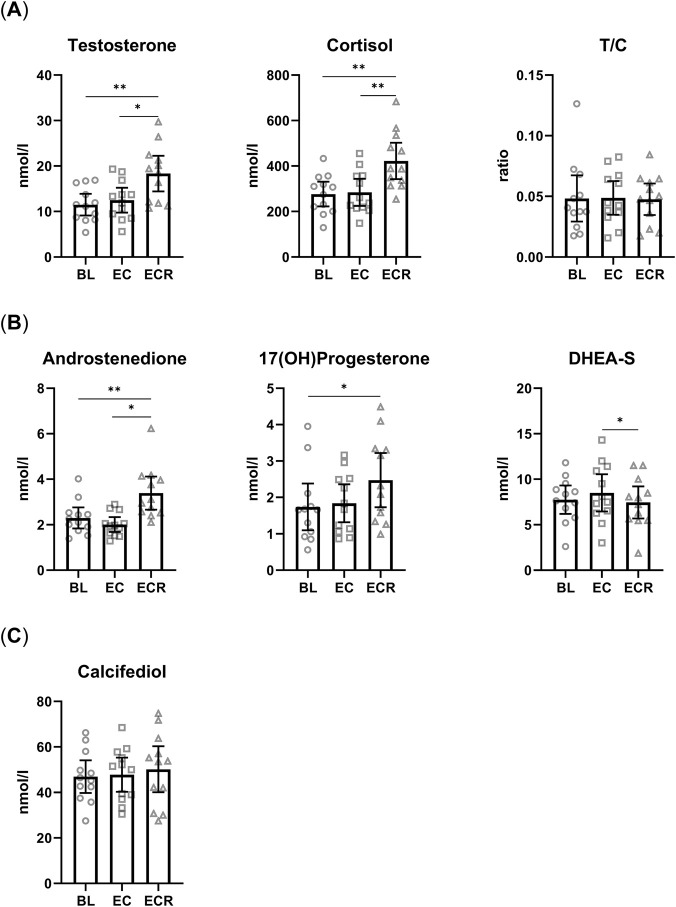
Effects of gradually increasing exercise and cold exposure on hormone levels. **(A)** Plasma concentrations of testosterone, cortisol, and the ratio of testosterone to cortisol (T/C) (n = 12). **(B)** Plasma concentrations of testosterone precursors androstenedione and DHEA-S, and the common precursor of testosterone and cortisol 17-OHP (n = 12). **(C)** Plasma concentration of calcifediol (n = 12). Baseline (BL), Exercise + cold (EC), exercise + cold recovery (ECR). Values are mean ± 95% CI; *p < 0.05, **p < 0.01. Repeated measures one-way ANOVA with Tukey’s multiple comparison test.

**FIGURE 2:**
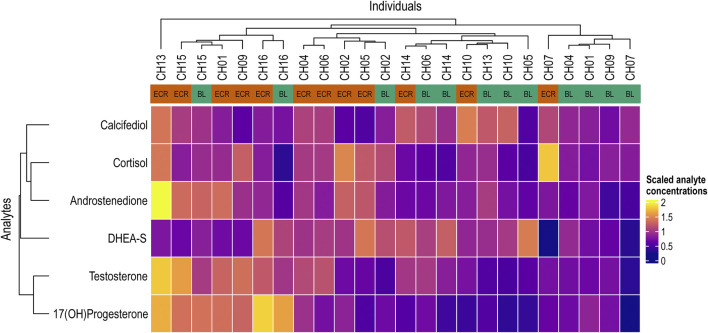
Heatmap of analysed steroid hormones. Heatmap of scaled values of all analysed hormones from each tested individual shows overall level differences between baseline (columns “BL”) and exercise + cold recovery (columns “ECR”).

In line with our main aim, we measured total serum testosterone levels and its precursors androstenedione and DHEA-S, we also measured the main stress hormone cortisol 17(OH)Progesterone which is precursor for both testosterone and cortisol, as well as calcifediol as a marker of overall vitamin D status (Figure 1). We did not observe any changes after EC compared to baseline across any of the hormones. However, after ECR we measured a significant increase in the levels of testosterone (+56%) and cortisol (+54%) compared to baseline (Figure 1A). They both followed a similar trend resulting in no changes to the testosterone to cortisol ratio (T/C) (Figure 1A). The increase in testosterone and cortisol was accompanied by an increase in testosterone precursor androstenedione (+48%) and their common precursor 17-OHP (+42%) after ERC compared to baseline (Figure 1B). Testosterone precursor DHEA-S showed an insignificant increase (+10%) post EC compared to baseline, which turned into a significant decrease after ECR (−13%) in comparison with post EC, essentially returning to baseline (Figure 1B). Calcifediol remained unchanged throughout the experiment (Figure 1C). Heatmap of scaled values allows us to visualize different measurements in a similar range (Figure 2). The heatmap shows overall hormonal changes between baseline and ECR. Apart from a couple of outliers, there is a clear separation between the two groups.

### 3.3 Analysis of SOD activity and inflammation markers after exercise in a cold environment

**FIGURE 3:**
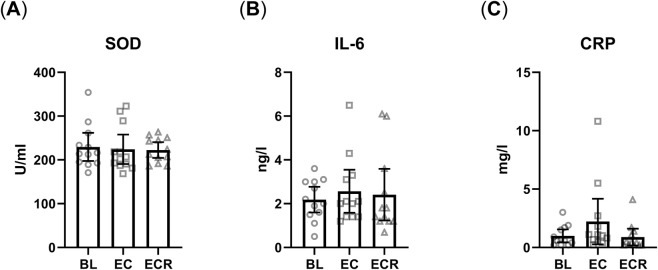
Circulating antioxidant and inflammatory parameters. **(A)** Enzyme activity of Cu/Zn superoxide dismutase (Cu/ZnSOD) in red blood cells. **(B)** Interleukin 6 (IL-6) and **(C)** C-reactive protein (CRP) concentrations in blood plasma. Baseline (BL), Exercise + cold (EC), exercise + cold recovery (ECR). Values are mean ± 95% CI. The Friedman test with Dunn’s multiple comparisons test was used.

Lastly, we aimed to assess systemic changes in general antioxidant and immune status. Thus, we measured the enzymatic activity of superoxide dismutase (Cu/ZnSOD) in erythrocytes (Figure 3A), and the blood plasma and serum concentrations of IL6 (Figure 3B) and CRP (Figure 3C), respectively (all graphs n = 12). One of the subjects IL6 peaked 0.1 above the reference level post EC but returned to normal after ECR. Likewise, 2 subjects expressed slightly elevated levels of CRP post EC but returned to normal after ECR. It is important to note that these subjects’ baselines were also significantly higher than the rest of the group, albeit still well within the normal range. Even with these outliers, we observed no significant changes across the experiment.

## 4 Discussion

This study revealed that 5-day exposure to cold during exercise does not affect hormone levels immediately. However, it significantly increases testosterone and cortisol levels for up to 7 days after the experiment. The unchanged testosterone levels at the end of the 5-day protocol align with Gagnon et al. (2014), who demonstrated that testosterone and cortisol levels do not change following low-intensity exercise in a cold environment. However, both cortisol and testosterone decreased slightly after moderate-intensity exercise in the cold compared to room temperature. Additionally, Solter and Misjak (1989) observed that cold exposure alone did not affect testosterone levels until −20 °C, at which point they reported a slight decrease. In contrast, Izawa et al. (2009) showed an increase in cortisol levels after exercise regardless of temperature and an interesting response in ice and in-line skaters who are accustomed to training in different temperatures. They demonstrated that even though exercising in 21 °C and 5 °C increased cortisol levels, the subjects experienced a greater increase in the temperature they were not accustomed to. Moreover, it has been shown that exercise can transiently increase circulating levels of cortisol depending on its intensity (VanBruggen et al., 2011). This may suggest a complicated dose-response relationship between exercise and cold. Their combination doesn’t automatically lead to altered levels of cortisol or testosterone and may be dependent on the prior status of the individual and the intensity of exercise and cold exposure.

The novelty of the present study lies in the simultaneous cortisol and testosterone increase in the post-exercise phase, i.e., 7-day recovery after the 5-day intensive training in a harsh cold environment. During the recovery period, the subjects returned to their day to day life no different from the baseline. It included both individual endurance and resistance training not exceeding the intensity of their training before or during the experiment and excluded cold exposure. We also excluded any unusual events or stressors during the recovery or immediately before the third blood sampling. The blood collections and InBody analysis were always done in the morning on an empty stomach. Since there were no changes after the 5-day intensive exercise in cold compared to baseline, it is unlikely that any regular exercise during the recovery phase would influence the last assessment.

The observed increase in total circulating testosterone was accompanied by an increase in its precursors, androstenedione and 17(OH)Progesterone. On the other hand, one of the precursors of androstenedione, DHEA-S, showed a slight decrease after recovery vs. post-experiment. We hypothesize that this could indicate upregulated synthesis of androstenedione from 17(OH)Progesterone in the biogenesis of testosterone (Yates and Deshpande, 1974). Additionally, we observed an increase in circulating cortisol levels, also supported by the increased 17(OH)Progesterone, a mutual precursor with testosterone (Held et al., 2020). To our knowledge, such a delayed effect of exercise or cold on increased testosterone or cortisol levels has never been published before. The longest observed period of elevated testosterone and cortisol was reported by Anderson et al. They showed that it took 48 h for cortisol and 72 h for testosterone to return to baseline levels after prolonged endurance exercise. However, unlike in our study, their values were elevated immediately after the exercise (Anderson et al., 2016).

The interaction between the hypothalamic–pituitary–adrenal axis and the hypothalamic–pituitary–gonadal axis is complex and makes the interpretation of concomitant increase in testosterone and cortisol challenging. One way of evaluating the relationship of these two hormones is the T/C. This ratio was historically used as a marker of anabolic/catabolic balance in the body, and its decrease was considered as a marker of overtraining (Mondal et al., 2025). However, current consensus suggests that T/C only indicates physiological strain dependent on the duration and intensity of exercise (Meeusen et al., 2013). Nevertheless, in our case, due to testosterone and cortisol increasing very similarly, their ratio did not change throughout the experiment. This suggests that no excessive acute or chronic stress was present. Moreover, an increase in cortisol does not necessarily invalidate the results, as cortisol was shown to be positively correlated with the increase in type II fibre area as well as the change in lean body mass after high-intensity resistance training (West and Phillips, 2012). As we already discussed, the literature associates cold exposure with transient testosterone decrease or blunted increase when combined with exercise (Sakamoto et al., 1991). A recent work by Gkika et al. (2020) showed that in both male and female rodents, testosterone improves mild cold (16 °C–20 °C) sensitivity via inhibition of mild cold receptor TRPM8. Testosterone does not act directly on TRPM8 but through a noncanonical activity of the androgen receptor, which does not affect TRPM8 expression. We speculate that this effect could result in an adaptive response. Moreover, we hypothesize that since it was repeatedly shown that cold acutely blunts testosterone levels, the adaptive increase might only happen later after a return to higher temperature. The existence of delayed adaptations is not a new notion. This has been mainly documented as changes in gene expression. For example, Neubauer et al. (2013) showed that after single endurance exercise trial in trained individuals the gene expression of hemoxygenase 1 significantly increased 3 h after exercise, remained increased after 48 h and reached peak expression 96 h after exercise. More interestingly McKay et al., (2008) showed that after single bout of intense resistance training mRNA levels of some members of the Insulin-like growth factor 1 family and myogenic regulatory factor 4 significantly increased only after 72 h and remained elevated 120 h after the exercise.

No changes in calcifediol are in line with some studies but contradictory to others. As mentioned in the introduction, acute endurance exercise can increase calcifediol levels (Mieszkowski et al., 2020; Dzik et al., 2022). However, Maïmoun et al., (2006) observed no changes in calcifediol after acute endurance exercise in competitive male cyclists. Effects of chronic endurance exercise seems to be tied to the baseline vitamin D status. While chronic endurance exercise was shown to increase calcifediol in people with deficiency (calcifediol <50 nmol/L) (Mieszkowski et al., 2018; Farag et al., 2019), it did not produce an increase in people with sufficient levels of calcifediol (>50 nmol/L) (Klausen et al., 1993; Mieszkowski et al., 2018). The mean baseline level of calcifediol for our subjects was 47 nmol/L which is just slightly under the threshold of 50 nmol/L considered to fulfill requirements for 97, 5% of the population (Ross et al., 2011). Moreover, exposure to sunlight has significant effect on vitamin D levels and many studies don’t specify this condition. Our study was performed in winter with the lowest amount of sunlight which may support our results. Therefore it is important to consider the baseline levels, nutrition and vitamin D supplementation as well as season when interpreting this data. Needless to say, none of the cited works combined exercise with cold exposure. Recently, CWI was shown to significantly increase the level of calcifediol in women with multiple sclerosis and to a lesser extent in healthy women, however no results in men have been published yet (Ptaszek et al., 2025).

Almost no changes in body composition may be explained by the group consisting of already very physicaly fit individuals used to multiple training sessions a week. The only significant change was measured in the waist to hip ratio (p = 0.049) after EC compared to baseline. This is supported by a slight decrease in visceral fat area. Gibas-Dorna et al. (2016) showed that cold water swimmer have higher body fat percentage, presumably for isolation, but lower visceral fat mass associated with lower insulin resistance.

Lastly, the activity of erythrocyte Cu/Zn SOD did not change, suggesting no lasting increase in erythrocyte oxidative stress and no improvement in the erythrocyte antioxidant defence, while anti-inflammatory IL-6 and general inflammation marker CRP exhibited only a marginal increase, due to a couple of outliers. It would be beneficial to expand on this and analyse a more complete panel of cytokines and antioxidants, which was unfortunately beyond the scope of this work.

In conclusion, this work highlights and begins to fill the gap in knowledge regarding the adaptive mechanisms during the late recovery phase after repeated exercise in a cold environment. Our novel results show a testosterone and cortisol increase without changes in their ratio in healthy male athletes 7 days after intensive exercise regimen combined with cold exposure.

### 4.1 Limitations of the study

We are aware of the limitations of this study, such as a small sample size and a limited number of blood draws and measured analytes. However, this is a pilot study, and considering the unexpected results, it should be repeated and expanded upon in the future. A further methodological limitation concerns the absence of a traditional control group. In the context of an intensive multi-day cold-exposure and exercise protocol performed in a remote mountain environment, establishing an external control group was not feasible. Individuals not undergoing the intervention would not be exposed to comparable environmental conditions, altitude, daily routine, or supervision, and creating a “sham” cold-exposure condition is inherently impossible. For these reasons, the study relied on a within-subject baseline (Day 0) as the reference point, using each participant’s initial physical status and hormonal profile as an individualized control. Although this approach is appropriate for interventions where a true control group cannot be implemented, it nonetheless limits causal interpretation. The observed endocrine responses must therefore be understood as changes relative to each participant’s pre-intervention state rather than differences between independent groups. Future studies may consider alternative comparative models—such as staggered interventions or partial-protocol control arms—to further refine causal inference.

## Data Availability

The original contributions presented in the study are publicly available. This data can be found here: https://figshare.com/articles/dataset/Increase_in_testosterone_and_cortisol_one_week_after_repeated_exercise_in_a_cold_environment/30019270.
